# Tunable methacrylated decellularized heart matrix: a versatile scaffold for cardiac tissue engineering

**DOI:** 10.3389/fbioe.2025.1579246

**Published:** 2025-06-12

**Authors:** Valinteshley Pierre, Douglas H. Wu, Chao Liu, Elif Ertugral, Chandrasekhar Kothapalli, Samuel E. Senyo

**Affiliations:** ^1^ Department of Biomedical Engineering, Case Western Reserve University, Cleveland, OH, United States; ^2^ Medical Scientist Training Program, Case Western Reserve University, Cleveland, OH, United States; ^3^ Department of Chemical and Biomedical Engineering, Cleveland State University, Cleveland, OH, United States

**Keywords:** decellularized heart matrix, biomaterials, ultra-violet crosslinking, methacrylation, matrix protein release

## Abstract

Therapeutic tissue regeneration remains a significant unmet need in heart failure and cardiovascular disease treatment, which are among the leading causes of death globally. Decellularized heart matrix (DHM) offer promising advantages for tissue engineering, including low immunogenicity and seamless integration into biological processes, facilitating biocompatibility. However, DHM is challenged by weak mechanical properties that limit its utility to biomedical applications like tissue engineering. To address this limitation, we functionalized DHM with methacryloyl functional groups (DHMMA) that support UV-induced crosslinking to enhance mechanical properties. By modulating the degree of methacryloyl substitution, a broad range of stiffness was achieved while maintaining cell viability on crosslinked DHMMA. Additionally, we show that increasing UV exposure time and pH increases DHMMA stiffness. Furthermore, topographical features transferred on DHMMA via soft lithography facilitated physical orientation of cells in culture. We demonstrate DHMMA as a scaffold with tunable stiffness and matrix-degradation properties suitable for cell survival and microfabrication for cardiac tissue engineering applications.

## 1 Introduction

Currently, there are no clinical therapies to repair the failing heart, motivating the need for advancements in cardiac tissue engineering strategies aimed at regenerating damaged myocardial tissue. The cardiac patch approach considers cell therapy on support scaffolds to rebuild parts of tissue (Wang Lu et al., 2021; Zhang et al., 2022). Synthetic biomaterials, such as poly (ε-caprolactone) and poly (lactic-co-glycolic acid), are used due to their tunable properties and facile synthesis (McMahan et al., 2020; Zhang Dongshan et al., 2024). Despite their versatility, synthetic biomaterials lack the complex biological cues for tissue integration and directed tissue repair (Kafili et al., 2023). Natural biomaterials comprised of gelatin, collagen, or decellularized ECM emerged as promising alternatives due to their biocompatibility (You et al., 2024; Xu et al., 2024). Specifically, there is a growing interest in exploring decellularized heart matrix (DHM) hydrogels as an alternative substrate for cardiac tissue engineering applications (Kafili et al., 2023; Vu et al., 2022; Zhe et al., 2023).

DHM offers a promising regenerative therapy for the heart due to the intrinsic properties of the native tissue microenvironment. DHM is comprised primarily of extracellular matrix proteins and provides insoluble building blocks such as collagen which suppress adverse tissue remodeling (McLaughlin et al., 2019; Baehr et al., 2020). DHM retains bioactive and soluble factors that are enriched in the cardiac tissue origin that facilitate crucial repair processes (Hamsho et al., 2024; Wu et al., 2023). The presence of cardiac tissue-specific ECM components facilitates programming of multicellular processes. When delivered to the ischemic heart, DHM promotes cardiomyocyte cell survival and proliferation, angiogenesis, suppresses fibroblast activation and potentiates immunogenic responses that support tissue repair (Jin et al., 2022). These collective effects help preserve cardiac function and reduce fibrosis (Wang et al., 2020; Wang et al. 2021a; Wang et al., 2021b; Wang et al. 2022; Diaz et al., 2021; Wassenaar Jean W. et al., 2016). We, along with others, have begun to elucidate the biofactors and responsive cellular players involved (Wang et al., 2021a; Wang et al., 2021b). Additionally, DHM has been explored as a delivery vehicle for matrix proteins and enriched soluble factors such as VEGF for vascularization (X. Wang et al., 2022). For cardiac therapy, DHM is delivered as a liquid hydrogel precursor. At physiological temperature, DHM undergoes gelation over several minutes (Wang et al., 2020; Wang et al. 2021a; Wang et al. 2021b; Wang et al., 2022). However, the slow gelation process presents a challenge for retention in the beating heart, with observable dispersion from the injection site. The spreading of DHM further accelerates degradation due to enzymatic remodeling processes. We previously developed solid DHM microparticles via electrospray and emulsification methods to increase retention after injection. The solid microparticles extended DHM tissue retention, increased resistance to enzymatic digestion, and promoted microvascularization compared to liquid DHM (Wang et al., 2022). Strategies to further improve long-term stability and control the physical properties of DHM will be critical for advancing both *in vitro* tissue modeling and clinical *in vivo* therapy.

Crosslinking and chemical functionalization are used to expand the limited material properties of natural biomaterials. Methacrylation has emerged as a pivotal technique to impart tunable physical properties to meet specific tissue engineering requirements. Methacrylation of ECM-derived biomaterials, such as gelatin (Shirahama et al., 2016; S. Chen et al., 2023; Yin et al., 2018), kidney (Ali et al., 2019) and liver decellularized matrix (Ravichandran et al., 2021), undergo ultra-violet (UV)-induced crosslinking, which introduces covalent bonds between polymer chains. This strategy allows for precise control over scaffold stiffness and degradation kinetics. In this context, exploring tunable crosslinking parameters, such as methacrylation buffer (Shirahama et al., 2016; S. Chen et al., 2023), UV exposure time (Choi et al., 2019), and pH (Shirahama et al., 2016) is essential for developing adaptable materials for biomedical applications. It has been demonstrated that increasing the degree of substitution of methacryloyl in gelatin (GelMA) increases its stiffness (Shirahama et al., 2016; Chen et al., 2023; Zhu et al., 2019). Methacrylation has yet to be explored with DHM hydrogels which provides a possibility for tuning the stiffness beyond simply changing the gel concentration.

In this study, we fabricated and characterized UV-crosslinkable DHM methacryloyl (DHMMA) with tunable mechanical and matrix degradation properties for cardiac tissue engineering applications. We demonstrated that the degree of methacryloyl substitution (DS) is dependent on the reaction buffer pH range. We showed that crosslinked DHMMA formed stable hydrogels that achieved a broad range of stiffness, controlled release of matrix proteins, and increased resistance to enzymatic digestion. Furthermore, we showed that the UV exposure time and pH of DHMMA suspension further tuned these properties. Additionally, we demonstrated that cardiomyoblasts remained viable and responded to physical alignment cues on crosslinked DHMMA. The tunable physical properties and biocompatibility of DHMMA makes it a promising candidate for *in vitro* modelling and *in vivo* therapeutic applications.

## 2 Materials and methods

### 2.1 Materials

Left ventricles came from adult Yorkshire/landrace porcine hearts (12–16 weeks). The following materials were used: Sodium dodecyl sulfate (SDS; Sigma Aldrich, Inc., United States), Triton X-100 (TX-100; Sigma Aldrich, Inc., United States), Pepsin (Sigma Aldrich, Inc., United States), Phosphate buffered saline (PBS; Research Products International, United States), Pepsin from porcine gastric mucosa (Sigma Aldrich, Inc., United States), 8N hydrochloric acid (HCl; Sigma Aldrich, Inc., United States), 8N sodium hydroxide (NaOH; Sigma Aldrich, Inc., United States), Methacrylate anhydride (MAA; Sigma Aldrich, Inc., United States), Irgacure 2959 (I2959; Sigma Aldrich, Inc., United States), Pure Methanol (Sigma Aldrich, Inc., United States), Polydimethylsiloxane (PDMS; Karyden, United States), High-glucose DMEM (ThermoFisher Scientific, Inc., United States), Penicillin-Streptomycin (P/S; ThermoFisher Scientific, Inc., United States), Fetal Bovine Serum (FBS; ThermoFisher Scientific, Inc., United States), TNBS (Sigma Aldrich, Inc., United States), Sodium bicarbonate (Sigma Aldrich, Inc., United States), and Gelatin Type A 300 bloom (Sigma Aldrich, Inc., United States).

### 2.2 Porcine heart decellularization and solubilization

Decellularized heart matrix (DHM) was generated based on our previous methods (Wang et al., 2019; Wang et al. 2021a; Wang et al. 2021a; Wang et al., 2022). Briefly, porcine cardiac tissue was harvested and prepared using protocols approved by Case Western Reserve University Institutional Animal Care and Use Committee (IACUC). First, an intramuscular injection of Telazol was used to anesthetize the pigs. The pigs were euthanized by an overdosage administration of Fatal-Plus, pentobarbital sodium (>100 mg kg^-1^). This method is based on recommendations by the 2000 Panel on Euthanasia of the American Veterinary Medical Association. Left ventricles, from 4 different pigs, were pulsed chopped in a food processor, washed with deionized water, and suspended in 1% SDS solution for 48 h with full decellularization indicated by a white color. The decellularized tissue pieces were then immersed in 1% TX-100 solution for 4 h. Tissues were subsequently washed 3 times in deionized water for 24 h then lyophilized. For solubilization, lyophilized DHM was cryo-pulverized and mixed with 0.01 M HCl, at 10 mg/mL, containing pepsin (1 mg/mL). The pH was lowered to 2-3 and the reaction proceeded for 48 h. The suspension was neutralized to pH 7.4, lyophilized, then stored at −80°C until further use. The decellularization protocol was adapted from previous protocol which confirmed removal of chromosomal DNA (Behmer et al., 2021; Williams, Sullivan, and Black, 2015; Singelyn et al., 2012).

### 2.3 Decellularized heart matrix-methacryloyl synthesis and chemical characterization

#### 2.3.1 Methacrylation

The lyophilized DHM was dissolved in either PBS or carbonate-bicarbonate (CB) buffer, both at pH 9.4, for 1 h using a modified protocol (Table 1) described elsewhere (Ali et al., 2019; Ravichandran et al., 2021). For DHM functionalization, methacrylic anhydride (MAA) was added dropwise (0.1 mL/min) to the DHM solution to achieve a final concentration of 2.5 mL/g MAA:DHM (in PBS or CB). The methacrylation reaction results in methacrylic acid as a byproduct, which lowers the pH of the DHM methacryloyl (DHMMA) suspension. To maintain basic pH, 1/6 of the total MAA volume required was added every 8 h after adjusting the reaction suspension to basic pH. All reactions proceeded for 48 h at 4°C under constant stirring. Then the suspension was neutralized (pH 7.4) and dialyzed against deionized water for 7 days using 5–7 K MWCO dialysis tubing, followed by lyophilization. Samples were stored dry at −80°C before use. Collectively, we achieved four DHMMA formulations: DHMMA synthesized in PBS or carbonate-bicarbonate buffer, with or without maintaining pH 9 ( $\text{DHMM}A^{\text{PBS}}$ ; $\text{DHMM}A^{\text{CB}}$ ; $\text{DHMM}A_{\text{pH}}^{\text{PBS}}$ ; $\text{DHMM}A_{\text{pH}}^{\text{CB}}$ ) presented in (Table 1).

**TABLE 1:** Method for producing the four formulations.

DHMMA	MAA [mL/g]	Buffer	pH 9 Maintained	Temp	Initial pH
PBS	2.5	PBS	No	4°C	9.4
CB	2.5	CB	No	4°C	9.4
PBS + pH	2.5	PBS	Yes	4°C	9.4
CB + pH	2.5	CB	Yes	4°C	9.4

GelMA was generated using the protocol detailed by Shirahama et al. (Shirahama et al., 2016; Luo et al., 2019). Briefly, gelatin was dissolved, under continuous stirring, in PBS or CB buffer at 50°C to a final concentration of 10% w/v. MAA was added to the gelatin solution dropwise (0.1 mL/min) to a final MAA concentration of 0.8 mL/g of MAA:Gelatin. A pH of 9 was maintained as 1/6 of the total MAA volume was added every 30 min. The reaction proceeded for 3 h at 50°C. GelMA was dialyzed, lyophilized, and stored dry at −80°C. Methacrylation was confirmed using TNBS assay (Supplementary Figure S1).

#### 2.3.2 Degree of methacryloyl substitution

The degree of substitution was determined using TNBS assay as described by Ali et al. (2019). Briefly, the four formulations of lyophilized DHMMA were resuspended in sodium bicarbonate solution at 1.6 mg/mL. Then 500 µL of 0.1% TNBS solution was added and allowed to react for 2 h at 37°C. The reaction was stopped by adding 500 µL of 10% SDS and 250 µL of 1N HCl. Optical density (OD) was measured at 340 nm on a plate reader (Bio-Tek Synergy H1 Hybrid Reader). The degree of methacrylation was calculated as follows:

$$Degree\;of\;Methacrylation=\left(1-\frac{OD\;of\;DHMMA}{OD\;of\;DHM}\right)\times 100$$

Chemical characterization of DHMMA was done by Fourier-Transform Infrared Spectroscopy (FTIR; Agilent 630). Approximately 5 mg of lyophilized DHM or DHMMA was placed in between the indenter and crystal of the instrument. The spectrum was set to 4000–600 cm^-1^ with step measurements of 4 cm^-1^. The background was calibrated before reading each group.

#### 2.3.3 Zeta potential

Zeta potential was measured in deionized water. Suspension of different formulations of DHMMA were diluted to approximately 1 mg/mL and injected into folded capillary zeta cell cuvettes. Measurements were taken using an Anton Paar Litesizer 500.

### 2.4 DHMMA crosslinking and physical characterization

#### 2.4.1 Crosslinking DHMMA hydrogel

DHMMA crosslinking was evaluated for all four formulations. Lyophilized DHMMA was suspended in deionized water at 20 mg/mL. To ensure full solubilization, the suspension was homogenized using Omni International TH-01 Homogenizer for 10 s. The pH was then adjusted to ∼7.4. After neutralization, photoinitiator (10% Irgacure 2959 stock in pure methanol) was mixed into the DHMMA suspension to achieve a final concentration of 0.5%. The mixture was then pipetted into PDMS molds (5 mm or 8 mm diameter discs). The molds were placed approximately 1 mm under the UV lamp (Spectroline Model ENF-240C) and exposed to 365 nm UV. The UV crosslinking time was 10 min unless otherwise indicated. Concentrations were based on values from the literature for ECM-based biomaterials (Ali et al., 2019; Ravichandran et al., 2021). The UV time was based on the intensity of the UV lamp which was approximately 2 mW/cm^2^ (Karl Suss, UV Intensity Meter Model 1,000).

The degree of DHMMA crosslinking was varied using two additional parameters: UV exposure time (30 s, 60 s, and 300 s) and pH (5, 7, 8, 9). The pH of the DHMMA suspension was adjusted before photoinitiator addition and UV crosslinking.

#### 2.4.2 DHMMA swelling

DHMMA (50 μL, pH 7.4) was crosslinked into 8 mm discs for swelling analysis. After UV exposure, the hydrogels were frozen at −80°C then lyophilized to normalize to the dry mass. The mass of the lyophilized samples was recorded, and the samples were immersed in 500 µL of deionized water. The swelling mass was measured after 24 h incubation at 37°C. The degree of swelling was calculated as follows:

$$Degree\;of\;Swelling=\frac{Mass_{wet}-Mass_{dry}\;}{Mass_{dry}}$$

#### 2.4.3 Mechanical compression of DHMMA

Mechanical compressive stress was measured using the Biomomentum MACH-1™ Mechanical Testing System (MA056-v500c) equipped with a 70 N load cell (MA235) and 8 mm diameter flat indenter. The measurements were performed on 50 µL of crosslinked DHMMA hydrogel with 5 mm diameter. Briefly, crosslinked DHMMA was loaded onto the sample stage. The indenter was initially lowered and upon contact with the surface of the gel started measuring the compressive stress as it displaced a total of 0.6 mm from the height of initial contact at a rate of 0.2 mm/s. The stress was obtained by retrieving the maximum force produced to compress crosslinked DHMMA and dividing it by the hydrogel cross-sectional area (5 mm diameter).

#### 2.4.4 Atomic force microscopy (AFM)

The DHMMA stiffness measurements were obtained with a MFP-3D-Bio Atomic Force Microscope (AFM; Oxford Instruments, Santa Barbara, CA, United States) at room temperature. Samples were generated as hydrogel discs (20 μL, 5 mm diameter) and were tested in triplicate. Modified tip-less AFM cantilevers (TL-CONT, Nanosensors; nominal spring constant: 0.02–0.77 N/m) with an 80-μm polystyrene bead were used and the actual spring constant was determined with a thermal calibration method before each experiment. Samples were placed on a Petri dish and force-distance curves were collected from random positions with a speed of 0.1 μm/s up to a setpoint of 2 nN in air. Four different sets of samples were analyzed, and Young’s Modulus (E_Y_) was calculated using the Hertzian model.

#### 2.4.5 Scanning electron microscope (SEM)

SEM (ThermoFisher Apreo2) was used to image the different formulations. All DHMMA formulations, at 20 mg/mL, were crosslinked for 10 min under UV light. The crosslinked DHMMA was cut in half prior to lyophilization to evaluate internal structure. Then, lyophilized DHMMA formulations were mounted on SEM stubs with carbon tape and sputter coated to 8 nm with iridium (Denton Desk V). To reduce charging effects and improve image quality, copper tape was used to ground samples to their respective stubs. Pore size distribution was analyzed using FIJI (v 2.16.0/1.54p). After cleaning text information from SEM micrographs, thresholds were applied using the Huang algorithm, and the “Analyze Particles” function was used. Thresholding and particle size limitations were adjusted to isolate surface layer pores or interior pores. Identified pore data was constructed into histograms using MATLAB (v 23a).

#### 2.4.6 Matrix-derived passive protein release

Protein release kinetics for all DHMMA formulations was determined by 30 days of incubation in neutral water. The four DHMMA formulations (50 µL) were crosslinked as described. The samples were lyophilized then weighed. The lyophilized DHMMA was placed in 500 µL of deionized water at 37°C. After the first day, the entire buffer was removed and stored, with fresh buffer replenishment every 3 days up to 30 days (infinite dilution). The aliquoted sample buffers were stored at −20°C. The Pierce™ BCA Protein Assay (ThermoFisher Scientific) was used to determine protein concentration by measuring absorbance at 540 nm using a plate reader (Bio-Tek Synergy H1 Hybrid Reader). Standard concentration curves were generated with all four DHMMA formulations to account for deviations in protein sensitivity due to the methacryloyl functionalization. Additionally, each gel was normalized to its initial dried mass.

#### 2.4.7 Collagenase digestion

DHMMA was subjected to enzymatic digestion for 24 h. The four DHMMA formulations (50 µL each) were crosslinked as described above. The crosslinked DHMMA was lyophilized, and the dry weight recorded. The digestion buffer was prepared by adding 1 mg/mL Collagenase Type I (Fisher Scientific) to 0.36 mM CaCl_2_. The lyophilized DHMMA was placed in 700 µL of digestion buffer and incubated at 37°C. Aliquots (45 µL) were removed, with replenishment, from the digestion buffer of the incubated gels at 5 min, 30 min, 1 h, 2 h, 4 h, 8 h, and 24 h. The aliquoted samples were stored at −20°C prior to analysis. The Pierce™ BCA Protein Assay (ThermoFisher Scientific) was used to determine protein concentration. Standard concentration curves were also generated for all four DHMMA formulations. Finally, each gel was normalized to its initial lyophilized mass.

### 2.5 Biocompatibility and biofabrication of DHMMA

#### 2.5.1 H9C2 cell culture

Crosslinked DHMMA was sterilized by immersion in 70% ethanol for 1 h followed by three washes with 1× PBS prior to cell seeding. H9C2 cells were cultured on crosslinked DHMMA or tissue culture plastic at 20,000 cells/well in a 96-well plate. Cells were maintained in high glucose DMEM containing 1% P/S, and 10% FBS. H9C2 cells were incubated for 72 h prior to analysis.

#### 2.5.2 Cell viability with live staining

Cell viability on crosslinked DHMMA was assessed using the Live/Dead Assay (ThermoFisher R37601). The four DHMMA formulations at 20 mg/mL were individually mixed with 0.5% Irgacure, poured into 5 mm diameter molds (15 µL), and exposed to UV. This generated crosslinked DHMMA hydrogels with 5 mm diameter and ∼1 mm thickness. For each DHMMA formulation, three biological replicates were produced, each with three technical replicates (averaged for each biological replicate). Cultured H9C2 cells were given fresh media mixed with Live/Dead solution (Calcein AM: BOBO-3 Iodide 1:1) and Hoechst (1 μg/mL) followed by 15 min incubation at room temperature. The hydrogels were removed from the wells to image the adhered H9C2 cells. Multispectral fluorescent images were captured using an Olympus IX81 fluorescence microscope, with three images taken per technical replicate to ensure representative sampling. Live cells (Calcein AM: green), dead cells (BOBO-3 Iodide: red), and total cell count (Hoechst: blue) were quantified using CellProfiler (version 4.25). Due to the high background intensity of DHMMA in the red channel, only the green channel for live cells was used for quantification of viability. Total cell count was determined from the Hoechst channel, while live cell counts were obtained by identifying overlapping objects in the Live and Hoechst channels. The percentage of live cells was calculated as the number of live cells divided by the total cell count.

#### 2.5.3 MTT assay

Metabolic activity of H9C2 cells on crosslinked DHMMA was assessed using the MTT assay (ThermoFisher). H9C2 cells cultured on crosslinked DHMMA were exposed to MTT for 4 h and the formazan crystals were dissolved with 10% SDS for 4 h. The neutralized solution was carefully removed and placed into a blank well to read the absorbance. Absorbance was read at 540 nm using a plate reader (Bio-Tek Synergy H1 Hybrid Reader).

#### 2.5.4 DHMMA micropatterning using soft-lithography

The master silicon and PDSM molds were fabricated following a previously described method (Watson et al., 2022; Liu et al., 2024). Briefly, a 100 mm silicon wafer (University Wafer, test-grade silicon) was spin-coated with SU-8 2025 photoresist (Kayaku Advanced Materials) to a thickness of 20 μm and patterned using a transparent glass photomask (Photo Sciences Inc.). After photolithography, the wafer was coated with a 500 nm layer of parylene C to facilitate PDMS demolding. PDMS molds were then cast from the silicon wafer, which featured grooves with four different widths (10, 20, 50, and 100 μm), using a standard single layer soft lithography technique. PDMS (Sylgard 184) base and curing agent (Dow 1317318) were mixed at a 10:1 (base-to-crosslinker) ratio, poured onto the wafer, and cured on a hot plate at 80 °C for 2 h. The resulting micropatterned PDMS mold was subsequently employed to create a negative template for the DHMMA micropatterned gel.

To imprint patterns on crosslinked DHMMA, two PDMS molds were utilized: one featuring micropatterns or a flat surface, and another open cylindrical mold with a 5 mm diameter placed atop to prevent spillover and ensure uniform gel height. The DHMMA suspension was carefully added onto the micropatterned or flat PDMS within the inner mold and exposed to UV light for crosslinking. Subsequently, solid gels were gently removed and transferred to tissue culture wells. For sterilization, the gels were immersed in 70% ethanol for 1 h. Following sterilization, micropatterned crosslinked DHMMA gels were rinsed 3 times with sterile PBS to eliminate residual ethanol.

#### 2.5.5 Optical profilometry

The topography of the micropatterned DHMMA, PDMS mold, and casting wafer surfaces was imaged using an optical profilometer (Zygo NewView 7300). The profilometer operated at ×5 and ×10 magnification, with measurement ranges of 1.04 mm × 1.05 mm and 0.71 mm × 0.53 mm, and resolutions of 2.19 μm and 1.10 μm, respectively. Data files were processed in Gwyddion (version 2.6.1) to measure the width and height of microgrooves and to generate 3D surface views.

#### 2.5.6 DHMMA microparticle generation using microfluidic chip

The PDMS microfluidic chips were fabricated using standard soft lithography techniques from a silicon wafer. The wafer contains eight replicates of three different nozzle sizes (10, 15, and 30 µm width), with one unit shown in Supplementary Figure S2. The PDMS base was mixed with a curing agent at a 10:1 ratio, poured onto the wafer, and baked at 80°C for 30 min. The inlets and outlets of the PDMS chip were then punched using a 1 mm biopsy punch (Electron Microscopy Sciences, United States, 69,039-10). Glass slides were spin-coated with PDMS mixed at a 20:1 ratio and partially cured for 12 min at 80°C. The PDMS chips were subsequently bonded to the PDMS-coated glass slides and baked for an additional hour.

The microfluidic chip was mounted on a light microscope and recorded using a camera connected to a computer, controlled via a microfluidic control system (Watson and Senyo, 2019). Tygon (McMaster-Carr, United States) tubing was inserted into the chip inlets and outlets using a bent stainless-steel needle after the material was loaded into the tubing with a syringe. Fluorinated oil containing 5% surfactant was introduced through the oil input. DHMMA was prepared at a concentration of 5 mg/mL and filtered to remove large aggregates, preventing channel clogging. To this solution, 0.5% Irgacure photo-initiator was added, and the solution was flowed through the water input. The DHMMA droplets were collected from the output and crosslinked under UV light using a UV lamp. The collected GelMA or DHMMA microparticles were dried in air, to evaporate the fluorinated oil, washed several times with ethanol, and stored in 1x PBS at 2°C–8°C.

#### 2.5.7 Immunostaining

Cells were fixed with 4% paraformaldehyde for 10 min at room temperature. The cells were permeabilized for 10 min (0.1% TX-100 in 1× PBS) and blocked for another 10 min (0.1% TX-100 + 5% Goat Serum in 1× PBS). Phalloidin-iFluor™ 555 (Cayman Chemical Company) was diluted 1:1,000 in the blocking buffer. The H9C2s were incubated in phalloidin for 45 min at room temperature. DAPI was added for 10 min. The wells were washed with 1× PBS and H9C2s imaged using Olympus BX60 Microscope with ×10 objective (Olympus UPlanFl 4/0.13).

### 2.6 Statistics

Graphs were generated using GraphPad Prism 8.0.1, and statistical analysis was conducted using one-way ANOVA with Tukey’s *post hoc* test. Statistical analysis for time dependent release curves was conducting using Two-Way ANOVA with repeated measurements. Error bars show the mean ± standard deviation (SD) for each group or mean ± standard error of the mean (SEM) for AFM analysis (significance shown by *p < 0.05, **p < 0.01, and ***p < 0.001, ****p < 0.0001).

## 3 Results

### 3.1 DHMMA synthesis and varying methacryloyl substitution

**FIGURE 1:**
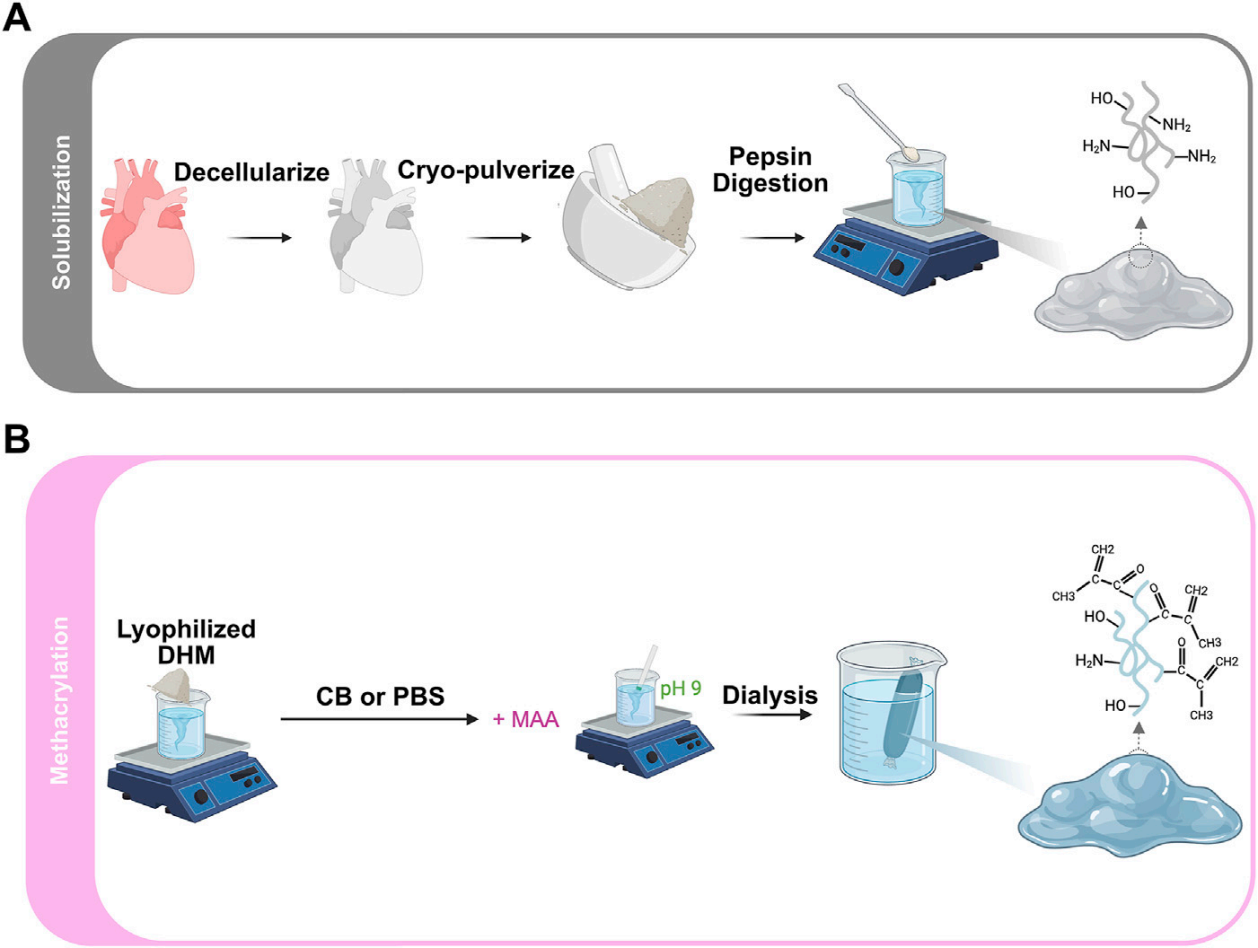
Schematic illustration of DHMMA fabrication process: **(A)** Decellularization, solubilization, and **(B)** Methacrylation.

**FIGURE 2:**
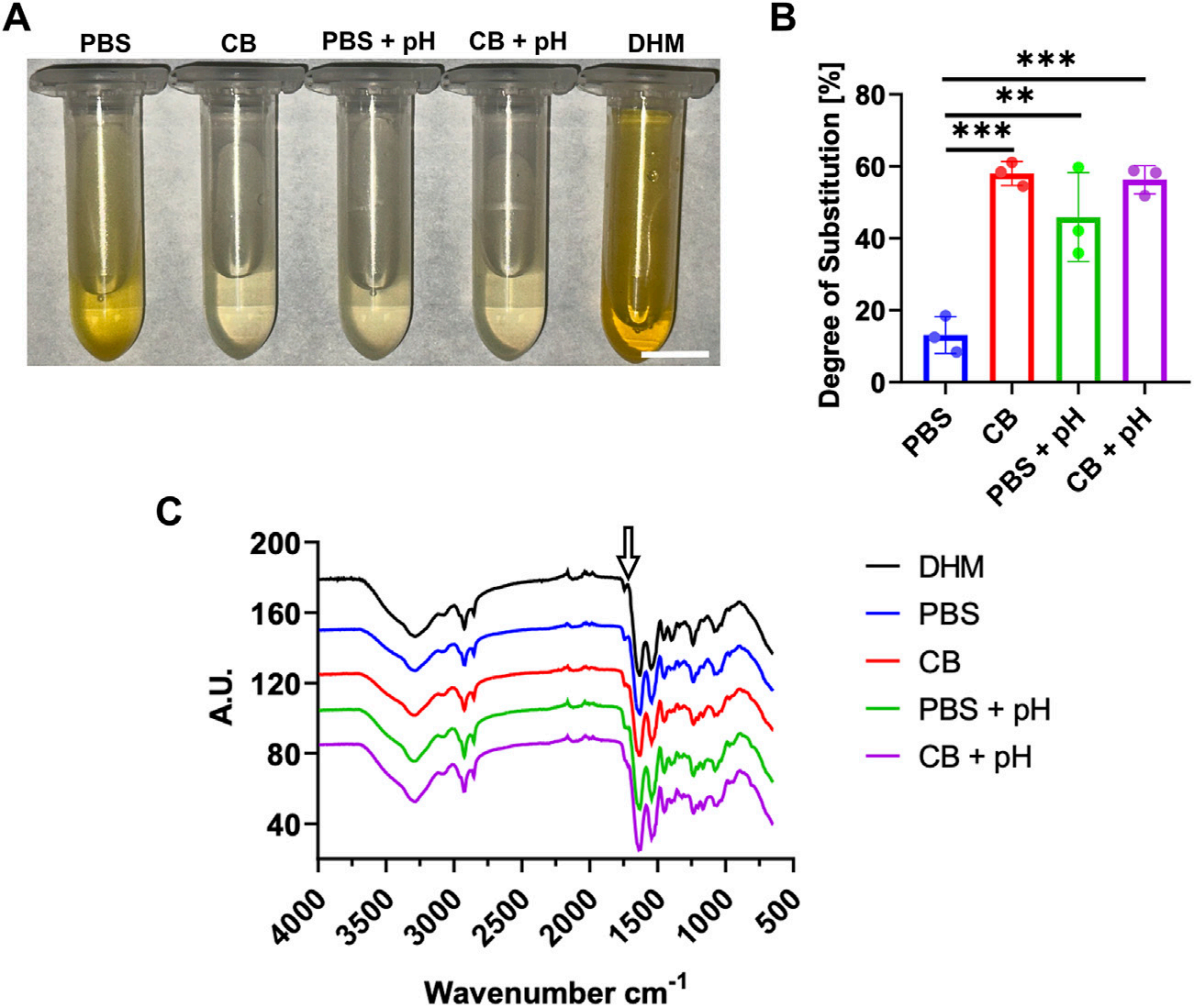
Chemical characterization of DHMMA. **(A)** Solutions of DHM/DHMMA from TNBS reaction to determine **(B)** degree of substitution. **(C)** FTIR spectra comparing DHM and DHMMA formulations. Arrow indicating the peak between 1720–1740 cm^-1^ increasing for all DHMMA formulations. Bar graph data represented as mean ± standard deviation, n = 3. Significance level: **p < 0.01, and ***p < 0.001. Scale bar = 10 mm.

The left ventricles of adult porcine hearts were decellularized using established protocols which we have previously shown to preserve core ECM components (Figure 1A) (Wang et al., 2021a; Wang et al., 2021a; Wang et al., 2022). The degree of substitution (DS) was varied by reacting methacrylic anhydride with DHM in PBS or CB buffer ( $\text{DHMM}A^{\text{PBS}}$ ; $\text{DHMM}A^{\text{CB}}$ ). Free methacrylic acid is produced as a reaction byproduct, decreasing the pH of the reaction solution below the optimal range for methacrylation (pH 8-9). To compensate, two additional groups were added where the pH was maintained at nine throughout the reaction time to achieve a higher DS ( $\text{DHMM}A_{\text{pH}}^{\text{PBS}}$ ; $\text{DHMM}A_{\text{pH}}^{\text{CB}}$ ) (Figure 1B). The DHMMA DS was quantified using the TNBS assay (Shirahama et al., 2016; Ali et al., 2019) (Figures 2A,B). The DS was calculated as the difference in light absorbance between DHM and DHMMA, normalized to DHM absorbance. Maintaining a basic pH throughout the reaction time resulted in a significant increase in $\text{DHMM}A_{\text{pH}}^{\text{PBS}}$ DS (45.9% ± 12.6%) compared to $\text{DHMM}A^{\text{PBS}}$ (13.1% ± 5.1%). Furthermore, methacrylation in CB buffer significantly increased the DS ( $\text{DHMM}A^{\text{CB}}$ , 58.0% ± 3.1%; $\text{DHMM}A_{\text{pH}}^{\text{CB}}$ , 56.3% ± 3.9%) compared to $\text{DHMM}A^{\text{PBS}}$ (13.1% ± 5.1%). However, maintaining a basic pH during methacrylation in CB buffer did not result in a significant change in $\text{DHMM}A_{\text{pH}}^{\text{CB}}$ DS (56.3% ± 3.9%) compared to $\text{DHMM}A^{\text{CB}}$ (58.0% ± 3.1%) (Figure 2B). During methacrylation, CB buffer maintained a stable basic pH due to its higher acid buffering capacity compared to PBS. The results suggest that DHMMA DS is increased in reaction buffers with greater acid buffering capacity. In PBS, maintaining a basic pH during methacrylation increases methacryloyl substitution. However, in CB buffer, adjusting the pH does not result in a significant increase in DS.

Methacryloyl substitution in DHMMA was further evaluated via Fourier-Transform Infrared Spectroscopy (FTIR) analysis (Figure 2C). FTIR spectra for DHMMA showed characteristic amide absorptions bands at 1,640 (C=C stretching) and 1,540 (N-H) cm^-1^, suggesting a retention of DHM structure with methacrylation. The increase in peak intensity at 1720–1740 cm^-1^ (C=O), relative to DHM spectra, suggests incorporation of ester and vinyl groups during methacrylation (Martineau, Peng, and Shek, 2005). Furthermore, a slight shift in the 1,540 cm^-1^ region suggests protein backbone modifications caused by methacrylate moieties. The data further supports that buffer choice and maintaining a basic pH influence methacryloyl substitution.

### 3.2 DHMMA crosslinking and physical characterization

The 20 mg/mL DHMMA concentration was used for subsequent experiments after it was determined in pilot studies that it retained the shape of the mold better than 10 and 5 mg/mL after crosslinking (Supplementary Figure S3).

**FIGURE 3:**
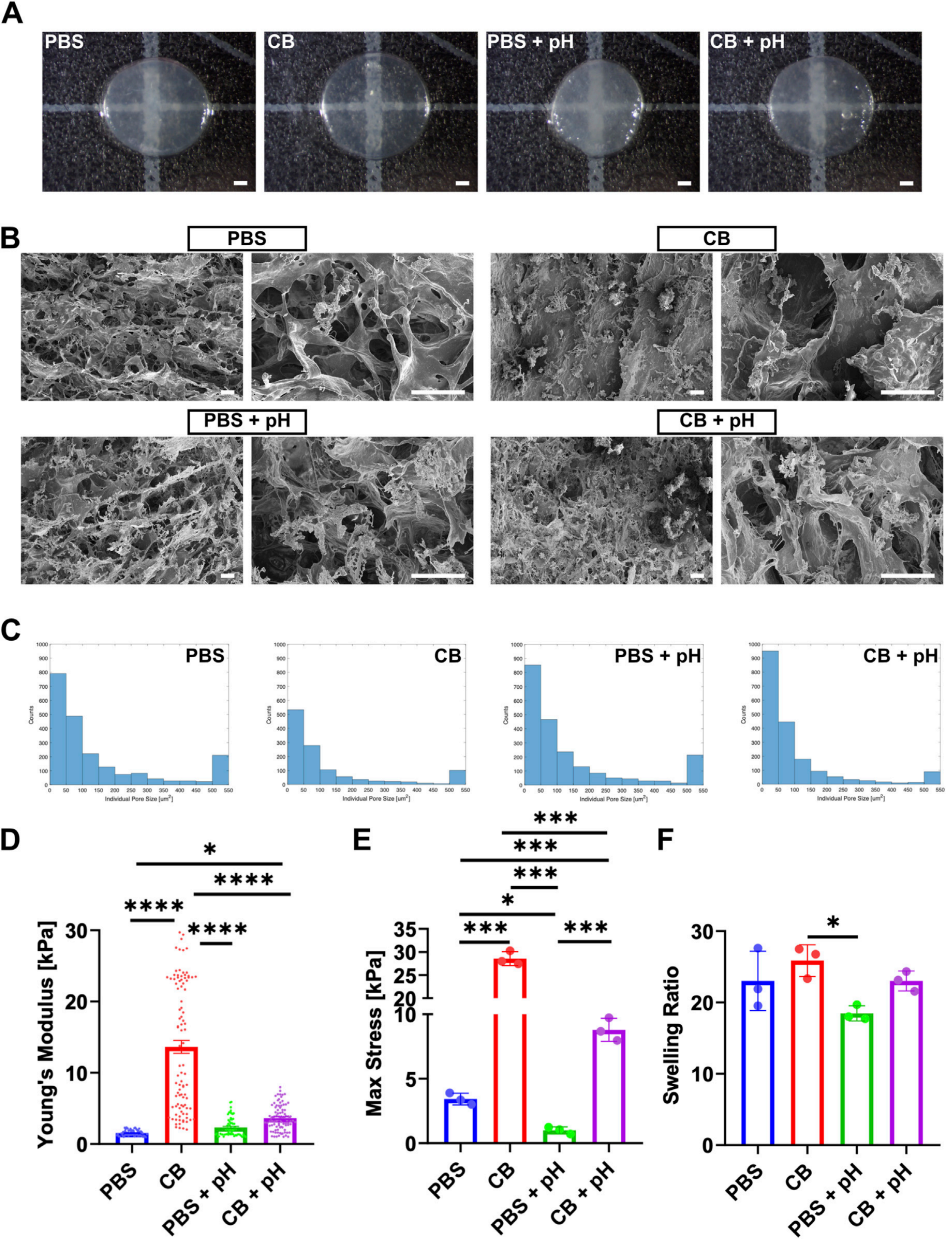
Physical characterization of crosslinked DHMMA. **(A)** Crosslinked DHMMA after 10 min of UV exposure (scale bar = 1 mm). **(B)** Representative SEM images at ×500 (left) and ×2000 (right) magnification (scale bar = 50 µm). **(C)** Histogram representation of changes in DHMMA cross-section with methacrylation reaction conditions. **(D)** Young’s Modulus of DHMMA from AFM analysis (mean ± standard error mean). **(E)** DHMMA compressed by 0.6 mm to determine the maximum stress. **(F)** Swelling ratio after immersion in DI water for 24 h at 37°C. All data represented as mean ± standard deviation, n = 3 gels. Significance level: *p < 0.05, **p < 0.01, ***p < 0.001, and ****p < 0.0001.

To determine the effect of reaction buffer conditions on DHMMA crosslinking, 50 µL of all four formulations of DHMMA was pipetted into 5 mm diameter PDMS molds and crosslinked. Visual inspection confirmed the crosslinking of all four formulations (Figure 3A). Crosslinked DHMMA was lyophilized, cut in half, and stored at −80°C. SEM imaging of the internal microstructure showed distinct differences across reaction conditions. Porosity analysis of the SEM images showed that DHMMA methacrylation in PBS resulted in more porous microstructure compared to methacrylation in CB buffer. More sheet-like structures were observed with $\text{DHMM}A^{\text{CB}}$ , with relatively fewer pores (Figures 3B,C). SEM analysis of the surface showed a similar trend (Supplementary Figure S4). In contrast, increased porosity is observed with pH adjustment in CB. Together, the results demonstrate that methacrylation buffer conditions alter DHMMA internal microstructure, with greater porosity in the PBS groups.

Mechanical properties were determined through AFM (local stiffness) and compression analysis (bulk stiffness). Measurement of local stiffness via AFM showed that methacrylation in CB buffer produced stiffer gels compared to PBS ( $\text{DHMM}A^{\text{CB}}$ , 13.63 ± 0.90 kPa; $\text{DHMM}A^{\text{PBS}}$ , 1.53 ± 0.04 kPa; mean ± SEM). There was a significant decrease in stiffness for $\text{DHMM}A_{\text{pH}}^{\text{CB}}$ (3.64 ± 0.18; mean ± SEM) compared to $\text{DHMM}A^{\text{CB}}$ (13.63 ± 0.90 kPa) (Figure 3D). The effect of methacrylation conditions on bulk mechanical properties was evaluated by compression analysis. Methacrylation in CB buffer significantly increased DHMMA stiffness ( $\text{DHMM}A^{\text{CB}}$ , 28.7 ± 1.5 kPa; $\text{DHMM}A^{\text{PBS}}$ , 3.4 ± 0.4 kPa). For reactions maintained at basic pH, a significant increase in stiffness was observed across buffers ( $\text{DHMM}A_{\text{pH}}^{\text{CB}}$ , 8.8 ± 0.9 kPa; $\text{DHMM}A_{\text{pH}}^{\text{PBS}}$ , 1.0 ± 0.3 kPa). However, within each reaction buffer, maintaining a basic pH did not increase the compressive modulus, rather it was significantly decreased (Figure 3E). Together this suggests that methacrylation in CB buffer increases DHMMA local and bulk stiffness. However, maintaining a basic pH during methacrylation decreases the stiffness.

Swelling is an intrinsic property of hydrogels that affect shape, mechanical properties, and small molecule diffusion. DHMMA swelling in water for 24 h was assessed by measuring the difference between the wet and dry mass, reflecting near-equilibrium water uptake. A significant increase in swelling ratio was observed for $\text{DHMM}A^{\text{CB}}$ (25.7% ± 2.2%) compared to $\text{DHMM}A_{\text{pH}}^{\text{PBS}}$ (18.5% ± 1.1%) (Figure 3F). Additionally, a more negative zeta potential was measured for high DS DHMMA (Table 2). Taken together, this data suggests that higher DS results in a more negative DHMMA which is achieved by methacrylation in CB buffer or maintaining a basic pH during methacrylation.

**TABLE 2:** Zeta potential of the 4 formulations of DHMMA.

DHMMA zeta potential		
Group	Mean zeta [mV]	SD
PBS	−20.84	0.62
CB	−34.53	1.50
PBS + pH	−36.53	1.43
CB + pH	−37.56	1.72

### 3.3 Matrix protein release and digestion

**FIGURE 4:**
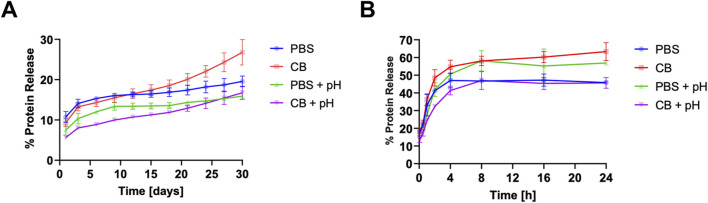
Passive and enzymatically induced matrix protein release. **(A)** Protein release from crosslinked DHMMA immersed in DI water at 37°C for 30 days with sampling every 3 days **(B)** DHMMA exposed to digestion buffer for 24 h at 37°C with sample aliquots taken at 5 min, 30 min, 1 h, 2 h, 4 h, 8 h, 16 h, and 24 h. Data represented as mean ± standard deviation, n = 3 gels. Statistics: Two-way ANOVA.

DHM contains cardiac-specific matrix protein that drive heart repair signaling (Hamsho et al., 2024; Jin et al., 2022; Bejleri and Davis, 2019). However, matrix protein release is challenged by rapid degradation *in vitro* and *in vivo* (X. Wang et al., 2022). To evaluate methacrylation effects on total protein release kinetics, crosslinked DHMMA was incubated in neutral buffer for 30 days at 37°C. All DHMMA formulations were crosslinked into 5 mm discs. A decrease in the percentage of matrix protein released with $\text{DHMM}A_{\text{pH}}^{\text{CB}}$ (Day 30: 16.7% ± 1.6%) compared to $\text{DHMM}A^{\text{CB}}$ (Day 30: 26.8% ± 3.1%) was observed. Only a few time points had a significant difference in percent protein release for $\text{DHMM}A^{\text{PBS}}$ and $\text{DHMM}A_{\text{pH}}^{\text{PBS}}$ (Figure 4A). Collectively, these findings suggest that maintaining a basic pH during methacrylation reduces passive matrix protein release.

Furthermore, all four formulations of crosslinked DHMMA were subjected to a 24 h collagenase (0.1 mg/mL) digestion. The mass of matrix protein released was significantly reduced for $\text{DHMM}A_{\text{pH}}^{\text{CB}}$ compared to DHMMA^CB^ after 24 h. For $\text{DHMM}A_{\text{pH}}^{\text{PBS}}$ , the mass of matrix protein released was significantly increased compared to $\text{DHMM}A^{\text{PBS}}$ (Figure 4B). This shows that the methacrylation conditions can alter matrix degradation of crosslinked DHMMA.

### 3.4 Varying UV crosslinking time

**FIGURE 5:**
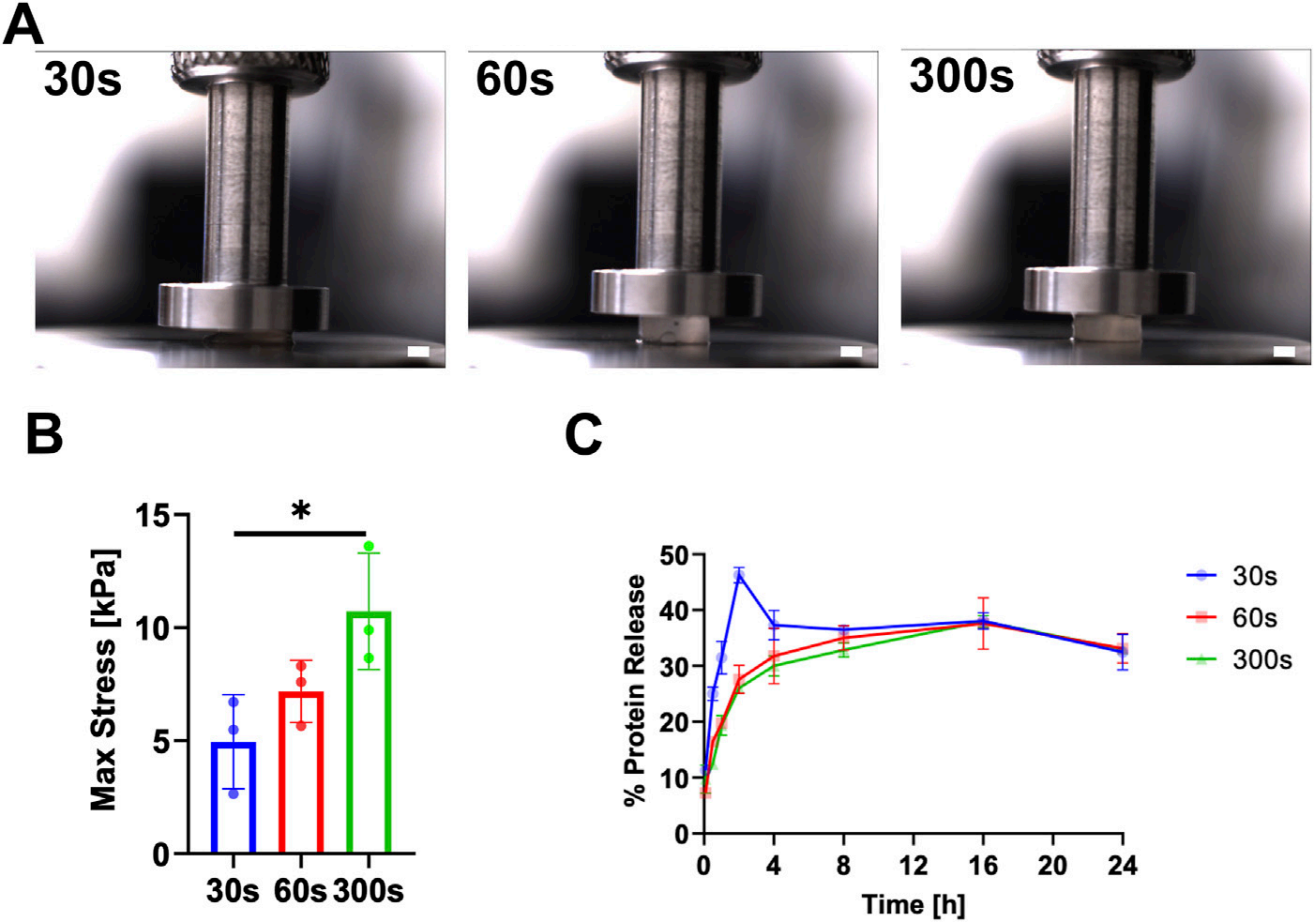
Physical characterization of DHMMA crosslinked for 30 s, 60 s, and 300 s. **(A)** Crosslinked $\text{DHMM}A_{\text{pH}}^{\text{CB}}$ compressed by 0.6 mm to determine **(B)** stiffness. **(C)** $\text{DHMM}A_{\text{pH}}^{\text{CB}}$ exposed to digestion buffer for 24 h at 37°C with sample aliquots taken at 5 min, 30 min, 1 h, 2 h, 4 h, 8 h, 16 h, and 24 h. All data represented as mean ± standard deviation, n = 3 gels. Significance level: *p < 0.05.

$\text{DHMM}A_{\text{pH}}^{\text{CB}}$ was chosen to determine the effects of UV crosslinking time due to its slow burst release within 4 h, making it suitable for drug release applications. The $\text{DHMM}A_{\text{pH}}^{\text{CB}}$ hydrogels were exposed to UV for 30 s, 60 s, and 300 s. There was a significant increase in stiffness from 30 s to 300 s UV (4.9 ± 2.1 kPa and 10.7 ± 2.5 kPa, respectively) (Figures 5A,B). As expected, this shows stiffness increasing with UV crosslinking time. Furthermore, $\text{DHMM}A_{\text{pH}}^{\text{CB}}$ crosslinked for 60 s and 300 s showed a lower percentage of matrix protein release with collagenase digestion for the first 4 h compared to 30 s (Figure 5C). The matrix protein release profiles for 60 s and 300 s were similar at all time points. The results suggest that increasing UV time can increase $\text{DHMM}A_{\text{pH}}^{\text{CB}}$ resistance to enzymatic stress within the first 4 h.

### 3.5 Varying DHMMA suspension pH

**FIGURE 6:**
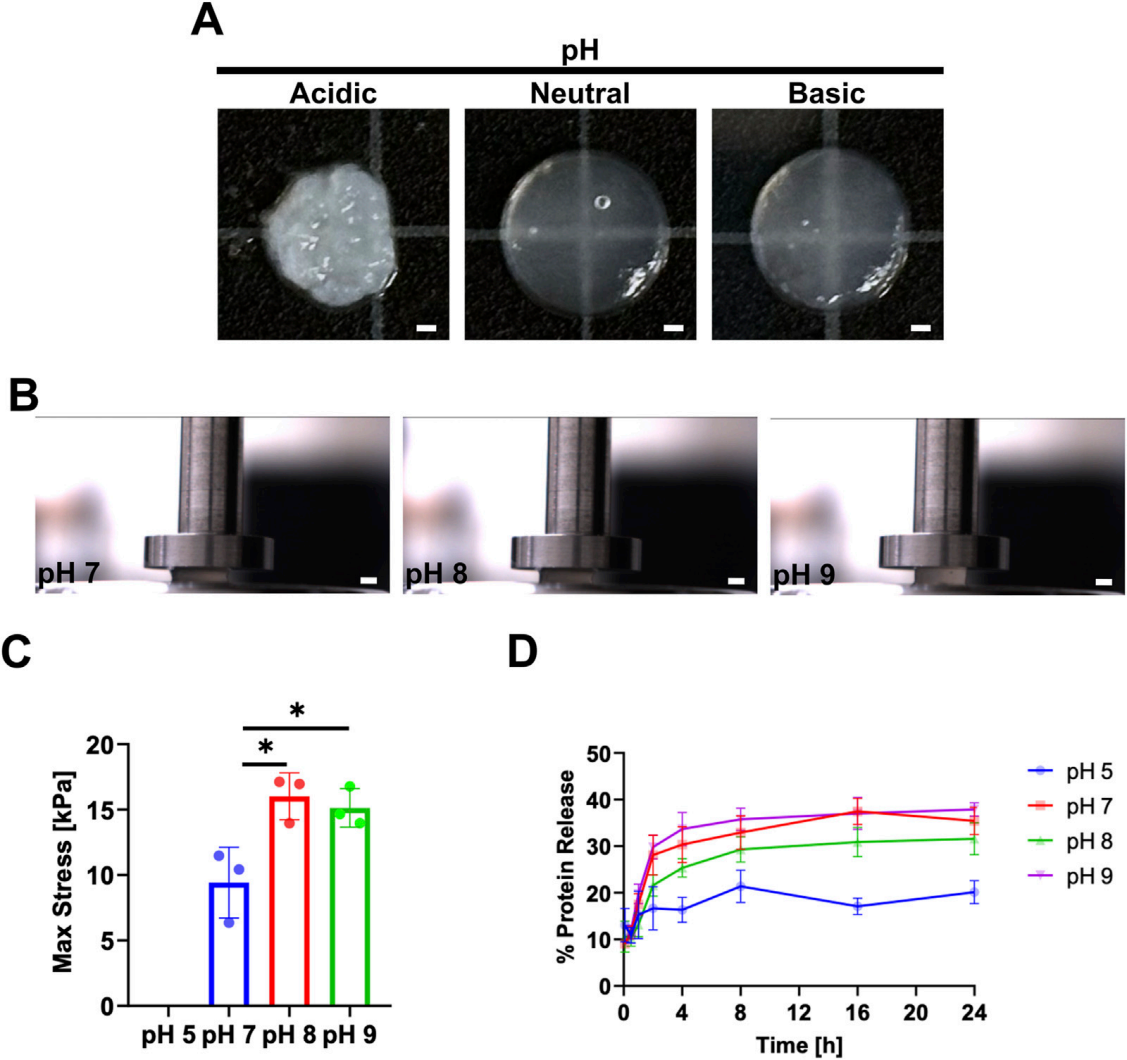
Varying DHMMA suspension pH prior to UV crosslinking. **(A)** Crosslinked hydrogels after adjusting $\text{DHMM}A_{\text{pH}}^{\text{CB}}$ suspension pH from acidic to basic. **(B)** Compression of $\text{DHMM}A_{\text{pH}}^{\text{CB}}$ after pH adjustment to determine **(C)** maximum stress (pH 5 was not solid enough to undergo compression). **(D)** $\text{DHMM}A_{\text{pH}}^{\text{CB}}$ after pH adjustment exposed to digestion buffer for 24 h at 37°C with sample aliquots taken at 5 min, 30 min, 1 h, 2 h, 4 h, 8 h, 16 h, and 24 h. All data represented as mean ± standard deviation, n = 3. Significance level: *p < 0.05. Scale bar = 1 mm.

To determine the effect of suspension pH on crosslinking, DHMMA pH was adjusted to 5, 7, 8, and 9 prior to UV exposure. $\text{DHMM}A_{\text{pH}}^{\text{CB}}$ prepared at acidic pH resulted in visible protein aggregates forming in suspension and unstable structures post-UV crosslinking (Figure 6A). This suggests that pH influences crosslinked $\text{DHMM}A_{\text{pH}}^{\text{CB}}$ stability.

To determine the effect of suspension pH on mechanical properties, $\text{DHMM}A_{\text{pH}}^{\text{CB}}$ prepared at pH 5, 7, 8, and 9 underwent compression (Figure 6B). A significant increase in $\text{DHMM}A_{\text{pH}}^{\text{CB}}$ stiffness at basic compared to neutral pH was observed (pH 7: 9.4 ± 2.7 kPa; pH 8: 16.0 ± 1.8 kPa; pH 9: 15.1 ± 1.4 kPa). However, there was no significant difference in stiffness between pH 8 and 9 (Figure 6C). The results show that adjusting the $\text{DHMM}A_{\text{pH}}^{\text{CB}}$ suspension to a basic pH increases its stiffness compared to neutral pH. Subjecting $\text{DHMM}A_{\text{pH}}^{\text{CB}}$ , prepared at pH 5, to collagenase digestion resulted in greater resistance compared to $\text{DHMM}A_{\text{pH}}^{\text{CB}}$ at pH 8 and 9 (Figure 6D). This suggests that although basic $\text{DHMM}A_{\text{pH}}^{\text{CB}}$ is stiffer, it is more susceptible to enzymatic digestion.

### 3.6 Cell behavior on crosslinked DHMMA

**FIGURE 7:**
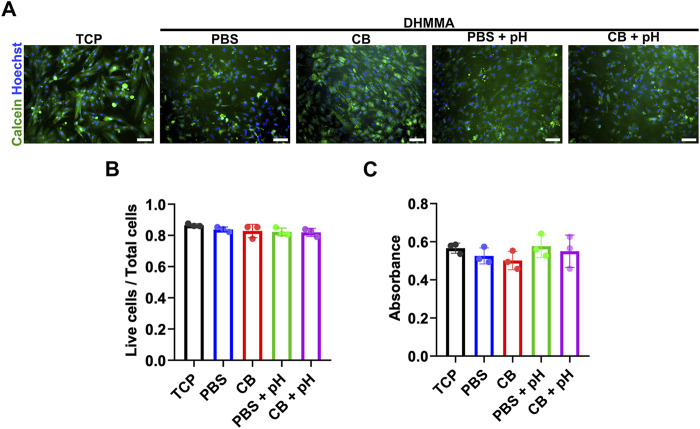
Live staining of H9C2 cells on crosslinked DHMMA. **(A,B)** H9C2 cells adhere and spread on crosslinked DHMMA and remain viable 72 h post-seeding. **(C)** MTT assay of H9C2 cells on crosslinked DHMMA after 72 h post-seeding. All data represented as mean ± standard deviation, n = 3. Significance level: ns (*p* > 0.05) for all conditions. Scale bar = 100 µm. TCP = tissue culture plastic.

Initial staining of H9C2 cytoskeleton on crosslinked $\text{DHMM}A^{\text{PBS}}$ show comparable cell morphology to control (Supplementary Figure S5A). Therefore, we proceeded with evaluating the impact of crosslinked DHMMA on H9C2 cardiomyoblasts viability using Live/Dead and MTT assay. H9C2 cells were cultured on crosslinked DHMMA discs (all four formulations) or tissue culture plastic (TCP) in maintenance media for 72 h. Then, the cells were stained and subsequently imaged (Figure 7A). Live cells were counted as the total number of nuclei stained green. There was no significant difference in the number of live cells across all groups, with each group maintaining greater than 80% viable cells (Figure 7B). The MTT assay further supported this, illustrating no significant differences in metabolic activity across all groups (Figure 7C).

Since methacrylation chemically modified DHMMA, we evaluated bioactivity after functionalization. H9C2 cells were exposed to soluble DHM or DHMMA (100 μg/mL) in growth media. Firstly, H9C2 cells cultured with soluble DHM or DHMMA for 24 h were labeled with BrdU for the last 4 h to determine proliferation frequency. No significant difference in H9C2 proliferation was observed across all groups (Supplementary Figures S5B, C).

### 3.7 DHMMA bio-fabrication using soft-lithography

**FIGURE 8:**
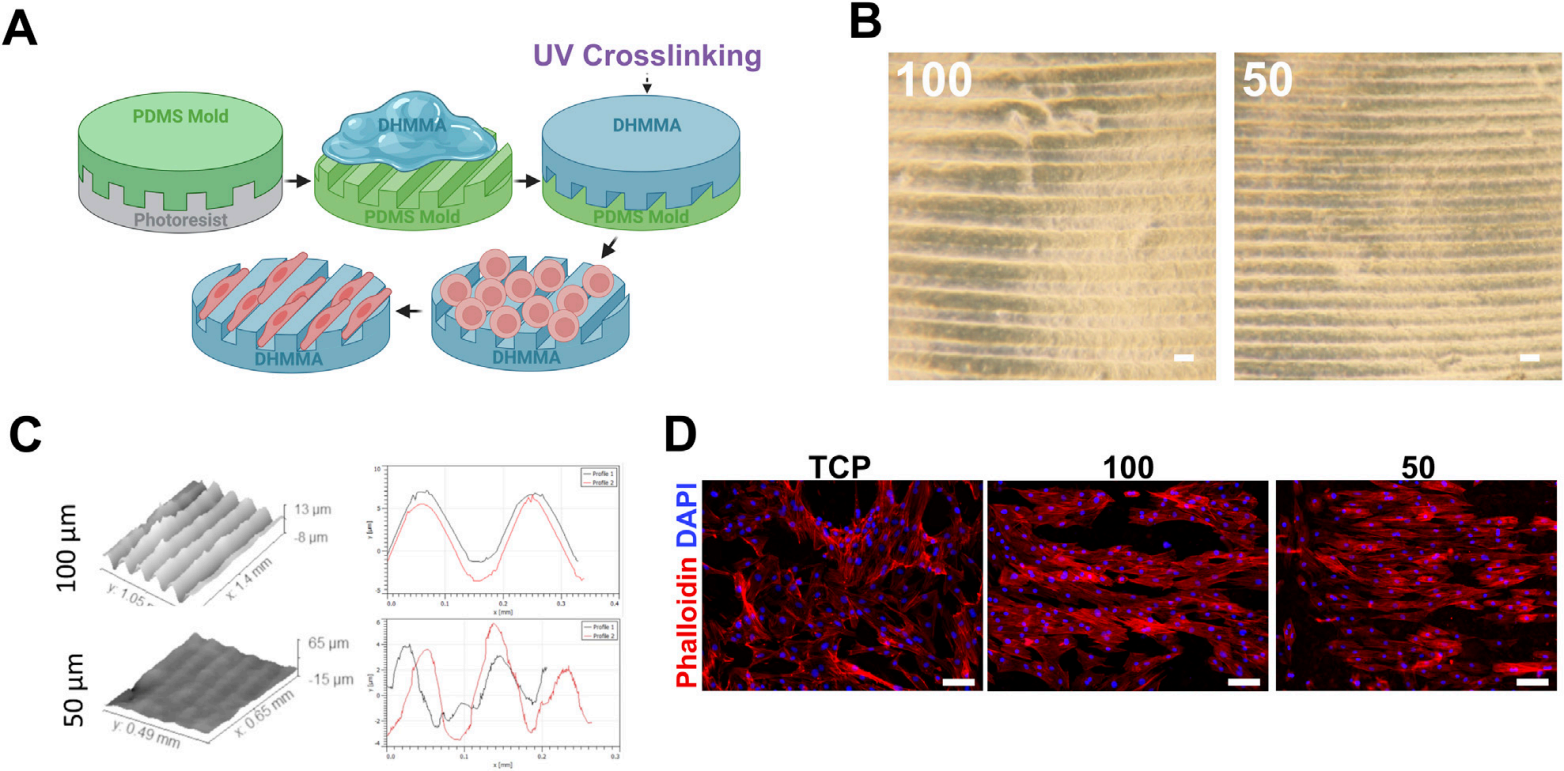
Crosslinked DHMMA for soft-lithography applications. **(A)** Fabrication process of micropatterned $\text{DHMM}A^{\text{PBS}}$ using soft-lithography. **(B)** Micropatterned $\text{DHMM}A^{\text{PBS}}$ . **(C)** Optical profilometry analysis of 100 μm and 50 µm groove patterns. **(D)** H9C2 cells aligning on micropatterned $\text{DHMM}A^{\text{PBS}}$ . Scale bar = 100 µm. TCP = tissue culture plastic.

The cardiomyocytes in the heart are highly organized. To support the use of DHMMA as a substrate for cell alignment, the softest formulation (based on AFM) was used to show the feasibility of surface micropatterning to promote anisotropic cellular organization. $\text{DHMM}A^{\text{PBS}}$ was crosslinked on PDMS molds containing 100 μm and 50 µm wide grooves (Figure 8A). Crosslinked $\text{DHMM}A^{\text{PBS}}$ was removed and micropatterns were verified using bright field microscopy and optical profilometry (Figures 8B,C).

Furthermore, the ability of micropatterned $\text{DHMM}A^{\text{PBS}}$ to align cells was evaluated by seeding H9C2 cells on the surface. Following overnight incubation, H9C2 cells were visualized using fluorescent microscopy after labeling their cytoskeleton with Phalloidin. Micropatterned $\text{DHMM}A^{\text{PBS}}$ with 100 and 50 µm features were effective in biasing alignment of H9C2 cells in the direction of the grooves (Figure 8D). This suggests that micropatterning $\text{DHMM}A^{\text{PBS}}$ confers additional functionality, enabling the promotion of uniform cell alignment, a crucial aspect for applications where cell polarity is essential for proper tissue function.

Other fabrication methods were employed including microparticle generation. Crosslinked $\text{DHMM}A^{\text{PBS}}$ microparticles were initially generated by immersing $\text{DHMM}A^{\text{PBS}}$ in mineral oil, with vortex, then UV crosslinked. Here we show generated $\text{DHMM}A^{\text{PBS}}$ and $\text{GelM}A^{\text{PBS}}$ microparticles with relatively homogenous morphology (Supplementary Figure S2A). Furthermore, we demonstrate a low polydisperse $\text{DHMM}A^{\text{PBS}}$ and $\text{GelM}A^{\text{PBS}}$ droplet population using microfluidics (Supplementary Figure S2B). In addition, we demonstrate tuning of droplet size by varying flow parameters (Supplementary Figure S2D). This supports the use of DHMMA as a versatile material for microparticle generation and potential drug delivery applications.

## 4 Discussion

DHM therapy for cardiac tissue repair faces challenges with maintaining its stability (resistance to mechanical forces) and durability (resistance to degradation) throughout the critical injury repair period in mammalian models of ischemic heart disease. While DHM hydrogels show promise in promoting cardiac repair, their rapid degradation and mechanical instability may limit their therapeutic efficacy (Shin et al., 2021). This research aims to address these limitations in cardiac tissue engineering by developing methacrylated DHM (DHMMA) with a broad range of stiffness and matrix protein release profiles. We and others have shown that injection of DHM in injured mice hearts induces cardiac repair (X. Wang et al., 2020; Wang et al. 2021a; Wang et al., 2021b; X. Wang et al., 2022; Diaz et al., 2021; Wassenaar Jean W. et al., 2016). Furthermore, previous attempts to enhance DHM stability through solid microparticle generation demonstrated increased tissue retention up to 2 weeks. However, since the repair period lasts up to 4–5 weeks (in murine models), there is still a need to increase the longevity of DHM (X. Wang et al., 2022).

While synthetic and composite hydrogels offer precise control over degradation and mechanical properties, they lack the complex protein milieu present in native cardiac ECM (Pisheh et al., 2024). In the heart, ECM composition changes with age (fetal to adult) and influence cardiomyocyte maturation (Cui et al., 2019; Derrick and Noël, 2021; Johnson et al., 2024). *In vitro*, delivery of ECM molecules promote cardiomyocyte proliferation (Bigotti et al., 2020; Sorbini et al., 2023), angiogenesis (Wang et al., 2021a; X. Wang et al., 2022), and fibroblast quiescence (Wang et al., 2021b). It is challenging for synthetic hydrogels to mimic the multicellular effects observed with native ECM. Therefore, there is a need to develop biomaterials that mimic the cardiac molecular composition. To match tissue stiffness, crosslinking using genipin, transglutaminase, or glutaraldehyde can stabilize ECM-derived biomaterials (Williams et al., 2015; You et al., 2024), but their rapid reaction kinetics severely limit fabrication methods such as 3D printing. Furthermore, challenges with toxicity limits practical use of some chemical crosslinkers (Jayachandran et al., 2022). Recent advances in ECM functionalization with methacryloyl functional groups suggest a promising direction for combining the advantages of natural and synthetic systems while maintaining biological complexity (Ali et al., 2019; Ravichandran et al., 2021; Behan et al., 2022).

### 4.1 Material properties and tunability

Here we demonstrate degree of substitution (DS) as a driver of DHMMA mechanical properties. A significantly greater DS was achieved in DHM methacrylated in CB buffer compared to PBS. Maintaining a basic pH (around 9) increases methacryloyl substitution in DHM. This is supported by results from the TNBS assay and FTIR analysis. There was no significant difference in DS across $\text{DHMM}A^{\text{CB}}$ , $\text{DHMM}A_{\text{pH}}^{\text{CB}}$ , and $\text{DHMM}A_{\text{pH}}^{\text{PBS}}$ based on the TNBS assay. We showed that DS was dependent on the reaction buffer, consistent with observations with GelMA (Shirahama et al., 2016). To the best of our knowledge, achieving different DS has not been published with decellularized matrix. We demonstrated that DHMMA methacrylation in CB buffer, as opposed to PBS, resulted in increased stiffness, likely due to increase in DS. This strategy demonstrated that we could achieve DS from 13% to 58% for DHMMA. Basara et al. successfully demonstrated that addition of GelMA and hyaluronic methacrylate (MeHA) to DHM allows for UV crosslinking and improve mechanical properties. This is further enhanced with transglutaminase crosslinking allowing them to achieve a stiffness range of 0.6–18 kPa (Basara et al., 2021). DHMMA significantly expands upon this range, achieving a compressive stiffness up to 28.7 kPa ( $\text{DHMM}A_{\text{pH}}^{\text{CB}}$ ). This broader range of stiffness is achieved due to the higher buffering capacity of CB buffer allowing for more methacryloyl substitution of amine residues on the proteins (Shirahama et al., 2016). This wide range covers tissue mechanics at developmental (1–3 kPa), healthy adult (8–17 kPa) (Y. Chen et al., 2020; Shin et al., 2021), and disease heart (>20 kPa) states.

We measured total protein release because matrix-derived proteins are hypothesized to be the key drivers of cardiac repair with injectable DHM therapy. The decellularization process preserves ECM components which stimulates heart repair. Using DHMMA, we evaluated how methacrylation affects total matrix protein release under both neutral and enzymatic buffer conditions. We found that maintaining a basic pH during methacrylation reduces total protein release in CB-buffered samples. For therapeutic applications, DHMMA thus enables a range of controlled release profiles. When combined with mechanical analysis, these findings provide insight into how best to engineer DHMMA to balance mechanical stability with the delivery of bioactive cues essential for cardiac repair.

Swelling capacity influences shape, mechanical properties, and molecule diffusion. We measured swelling over 24 h based on established findings that DHM swelling typically stabilizes within this timeframe (X. Wang et al., 2022). The lack of trend observed with DHMMA swelling may be due to counteracting factors such as protein heterogeneity, size, conformation, DS, charge (Panahi and Baghban-Salehi, 2019; Nichol et al., 2010). Only some of these factors are measured in this study. Further investigation is needed to understand the degree of contribution to matrix swelling. The increase in DHMMA zeta potential with methacrylation is consistent with what is observed with collagen (Yang et al., 2020) and may promote cell survival (Y. M. Chen et al., 2009). Although lyophilizing samples before rehydration differs from fully hydrated physiological conditions, it reflects the scenario of prepackaged, dried scaffolds that subsequently swell upon implantation to conform to the target site. From a tissue engineering perspective, the ability to affect swelling behavior offers a strategy to balance mechanical strength with sufficient porosity and diffusivity for cells and bioactive molecules. Therefore, optimizing methacrylation may provide a practical avenue to tailor structural and functional properties of DHMMA for cardiac repair applications.

UV exposure time emerged as a critical factor in fine-tuning the final mechanical properties of crosslinked DHMMA at constant concentration. Typically, the stiffness of a hydrogel is enhanced by increasing protein concentration. Varying protein concentration can achieve a wide range of stiffness for hydrogels such as GelMA (around 1–80 kPa, 2.5%–10% w/v) (O’Connell et al., 2018). However, the increased protein density may hamper cell functionality, especially in 3D where they adopt a spherical morphology due to decrease rate of stress relaxation (Shin et al., 2021; Chaudhuri et al., 2015; Shie et al., 2020; He et al., 2023). Building on the mechanical control achieved through methacrylation, we confirmed UV exposure time as an additional parameter for tuning mechanical properties at constant concentration. We observed enhanced $\text{DHMM}A_{\text{pH}}^{\text{CB}}$ stiffness with increasing UV exposure time, supporting the literature. Greater resistance to enzymatic stress, within the first 4 h, was achieved with longer UV exposure time. While UV crosslinking for 60 s and 300 s had comparable release kinetics under enzymatic stress, 60 s UV $\text{DHMM}A_{\text{pH}}^{\text{CB}}$ broke into large fragments within 8 h compared to 300 s UV $\text{DHMM}A_{\text{pH}}^{\text{CB}}$ . This shows the suitability of DHMMA as a drug delivery tool to achieve ‘fast’ or ‘slow’ protein release. This may eliminate the need for multiple therapeutic injections by generating a heterogeneous population of ‘fast’ or ‘slow’ releasing biomaterials to release payload during early and late stages of injury repair.

We observed that the DHMMA suspension pH, prior to UV exposure, offered an additional layer of tunability. First, we observed increase in $\text{DHMM}A_{\text{pH}}^{\text{CB}}$ suspension viscosity with increasing pH (∼5–8). Crosslinking $\text{DHMM}A_{\text{pH}}^{\text{CB}}$ suspension prepared at pH 5 resulted in a material too soft for mechanical compression analysis. $\text{DHMM}A_{\text{pH}}^{\text{CB}}$ at pH 8 and 9 were significantly stiffer than pH 7. This is consistent with observations showing that UV-initiated riboflavin crosslinking of collagen was stiffer at basic pH (range 8–9) than pH 7 (Diamantides et al., 2017). They also showed that stiffness peaks at pH eight and decreases with pH > 8. Similar trends were observed with gelatin and collagen (Kim and Bonassar, 2023; Goudie et al., 2023) although with shifts in peak stiffness likely due to differences in isoelectric points. Contrary to what was expected, the softer pH 5 $\text{DHMM}A_{\text{pH}}^{\text{CB}}$ showed the least amount of matrix protein release when exposed to collagenase. Research shows that collagen fibril dimensions and organization are pH dependent. Thicker collagen fibrils are formed at acidic pH and may contribute to slower enzymatic breakdown. In contrast, collagen in basic pH form thinner fibrils which may make them more susceptible to enzymatic activity (Roeder et al., 2002). Although collagen and gelatin are suitable models to approximate the effects of pH on DHMMA, due to its high collagen content (Hamsho et al., 2024), the heterogenous composition of DHMMA warrants further characterization.

This tunable approach addresses a key limitation of ECM-hydrogels from decellularized tissue: stiffness less than 1 kPa. Achieving a broad range of mechanical properties at a constant protein concentration represents a significant advancement.

### 4.2 Structural characteristics

The fabrication parameters we investigated not only influenced mechanical properties but dramatically impacted the microscale architecture of crosslinked DHMMA. Varying the methacrylation reaction conditions resulted in distinct structural features suited for different applications.

Hydrogel porosity plays an important role in facilitating bulk erosion, cell, and nutrient infiltration (Xiang and Cui, 2021). Controlling the crosslinked DHMMA porous microstructure by varying fabrication parameters has significant implications for material function. SEM analysis revealed that PBS-mediated methacrylation produced highly porous structures ideal for cell infiltration and tissue integration, while CB buffer yields sheet-like structures better suited for controlled drug delivery. This structural versatility provides unique control over microstructure eliminating the need of a sacrificial layer. The degree through which microstructure can be tuned by varying other crosslinking parameters remains to be determined. The ability to control both bulk properties and microscale features represents a significant advantage over traditional DHM crosslinking methods, where structural control is often limited.

### 4.3 Biological performance

While methacrylation expands the physical properties of a biomaterial, the impact on cell viability is crucial for tissue engineering adoption. DHMMA demonstrates excellent biocompatibility across all four formulations, with H9C2 viability greater than 80%. Furthermore, no significant difference in metabolic activity was observed between all groups. These results are comparable to cell viability on other crosslinked ECM-derived hydrogels and suggest minimal impact of methacrylation on cellular biocompatibility with no visual evidence that suggest cell death (Ferchichi et al., 2024). While 3D cell encapsulation remains to be evaluated, the initial data suggests minimal impact of crosslinked DHMMA on cellular compatibility. This is likely due to the retention of sufficient cell adhesion sites available since the highest DS achieved is ∼58%. For 3D cell encapsulation, cell viability is negatively impacted by UV exposure time (Pepelanova et al., 2018; Ding, Illsley, and Chang, 2019). This can be mitigated by using higher UV intensity with shorter crosslinking time (<60 s) or a visible light-activated photoinitiator (Choi et al., 2019). Soluble DHMMA had no significant impact on H9C2 proliferation *in vitro* suggesting maintained viability and comparable response to previously established DHM (Wang et al., 2020). This suggests that methacrylation of DHM has minimal impact on biocompatibility. Future work will evaluate different DHMMA dosages on cardiac cell behavior.

### 4.4 Applications

Micropatterning allows for precise control over substrate topography, which is essential for guiding cell behavior (e.g., maturation) (Zhang Bin et al., 2024) and promoting tissue organization in cardiac tissue engineering (Y. Zhang et al., 2022). The successful transfer of micropatterns and subsequent cellular alignment illustrates the potential of DHMMA to facilitate biomimetic microenvironments conducive to cell and tissue organization. We demonstrated successful pattern transfer on the softest DHMMA formulation determined by AFM analysis ( $\text{DHMM}A^{\text{PBS}}$ ) and cell alignment in the groove direction. This demonstrates that in addition to providing native biological cues, DHMMA can be fabricated to provide physical cues beyond stiffness, such as alignment. The ability to achieve aligned cellular organization through topographical guidance particularly addresses the critical need for proper cardiomyocyte orientation in engineered cardiac tissues. The effect of alignment on cardiac biology is a well-accepted concept and future studies will evaluate the impact *in vivo* (Zhang et al., 2024a; Kalkunte et al., 2023; English et al., 2023).

We previously showed that generated solid DHM microparticles resulted in increased tissue retention post-injection. Furthermore, microparticles were stiffer and showed greater resistance to enzymatic stress (Wang et al., 2022). While DHM microparticles were beneficial to heart repair, electrospray microparticles had relatively high polydispersity. Furthermore, thermal gelation only achieved particle retention up to 2 weeks. We hypothesized that crosslinking will further enhance the longevity of DHM and a more homogenous population can be achieved using droplet microfluidics (Moreira et al., 2021). Our results show that generated DHMMA microparticles via water-in-oil emulsion (bulk and microfluidics methods) had homogenous morphology. Moreover, we demonstrate that droplet size can be controlled by varying flow parameters. The ability to generate microparticles with distinct size and tunable physical properties potentiates DHMMA as a drug delivery biomaterial for cardiac tissue engineering applications.

### 4.5 Limitations and future directions

While UV-induced crosslinking offers numerous advantages, including rapid processing and spatiotemporal control over hydrogel properties, several limitations warrant consideration. The cytotoxicity of UV radiation and the potential for photoinitiator residues pose challenges to embedded-cell viability and scaffold biocompatibility for future 3D culture studies. Moreover, achieving homogeneous crosslinking throughout the heterogeneous DHMMA remains a critical aspect to ensure consistent stability and durability. The heterogenous protein composition of DHMMA makes it challenging to characterize due to varying DS for different proteins. Furthermore, different matrix proteins have different charges, isoelectric points, and solubility amongst other properties. Chemical characterization such as NMR is challenged by insoluble DHMMA components. Despite this, it is likely that we will observe similar but not identical behavior between GelMA, collagen, and DHMMA.

Moving forward, addressing these limitations requires a comprehensive understanding of ECM-derived methacrylate hydrogel fabrication, including chemical characterization, optimizing crosslinking parameters, and evaluating biocompatibility profiles. Additionally, elucidating the influence of methacrylation on ECM-derived hydrogel bioactivity and its impact on cellular behavior is paramount for advancing cardiac tissue engineering strategies. These limitations, while significant, point to clear directions for future development that could further enhance DHMMA utility in cardiac tissue engineering.

## 5 Conclusion

The development of DHMMA represents a significant advancement in cardiac tissue engineering biomaterials, providing control over physical properties by tuning DS (indirectly), UV exposure time, and pH. This versatility enables a broad range of mechanical properties matching native cardiac tissue, from developmental to disease states, while preserving the inherent biological complexity of the ECM. Furthermore, DHMMA fabrication parameters allow regulation of microscale architecture achieving varying degrees of porosity suitable for different applications. Cell viability and alignment on crosslinked DHMMA provides a rationale for investigating DHMMA as a viable option for cardiac tissue engineering applications.

## Data Availability

The original contributions presented in the study are included in the article/Supplementary Material, further inquiries can be directed to the corresponding author.
