# Supplementary material for: Tunable methacrylated decellularized heart matrix: a versatile scaffold for cardiac tissue engineering
Source: Front Bioeng Biotechnol. 2025 Jun 12;13:1579246. doi: 10.3389/fbioe.2025.1579246 (PMC12198208; doi:10.3389/fbioe.2025.1579246)
Supplement: Supplementary file 1 [file DataSheet1.docx]

Supplementary Material

# Supplementary Figures


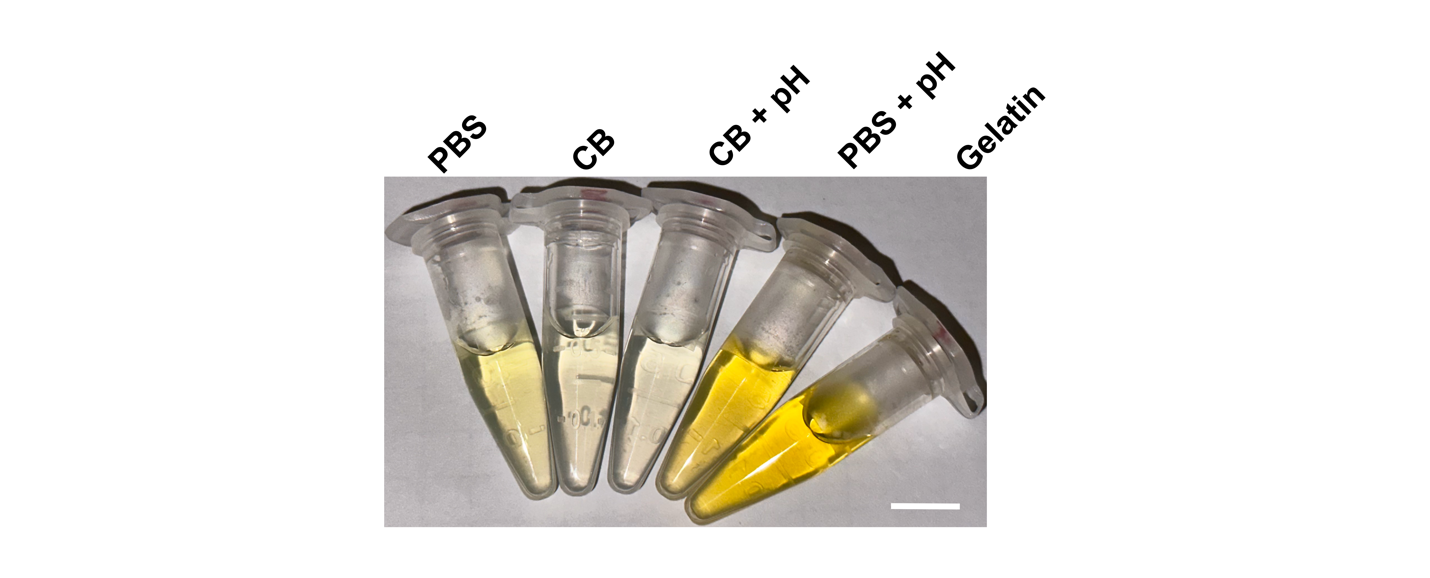


**Figure S1.** TNBS performed on GelMA methacrylated under 4 different conditions with gelatin as a control. Scale bar = 10 mm.


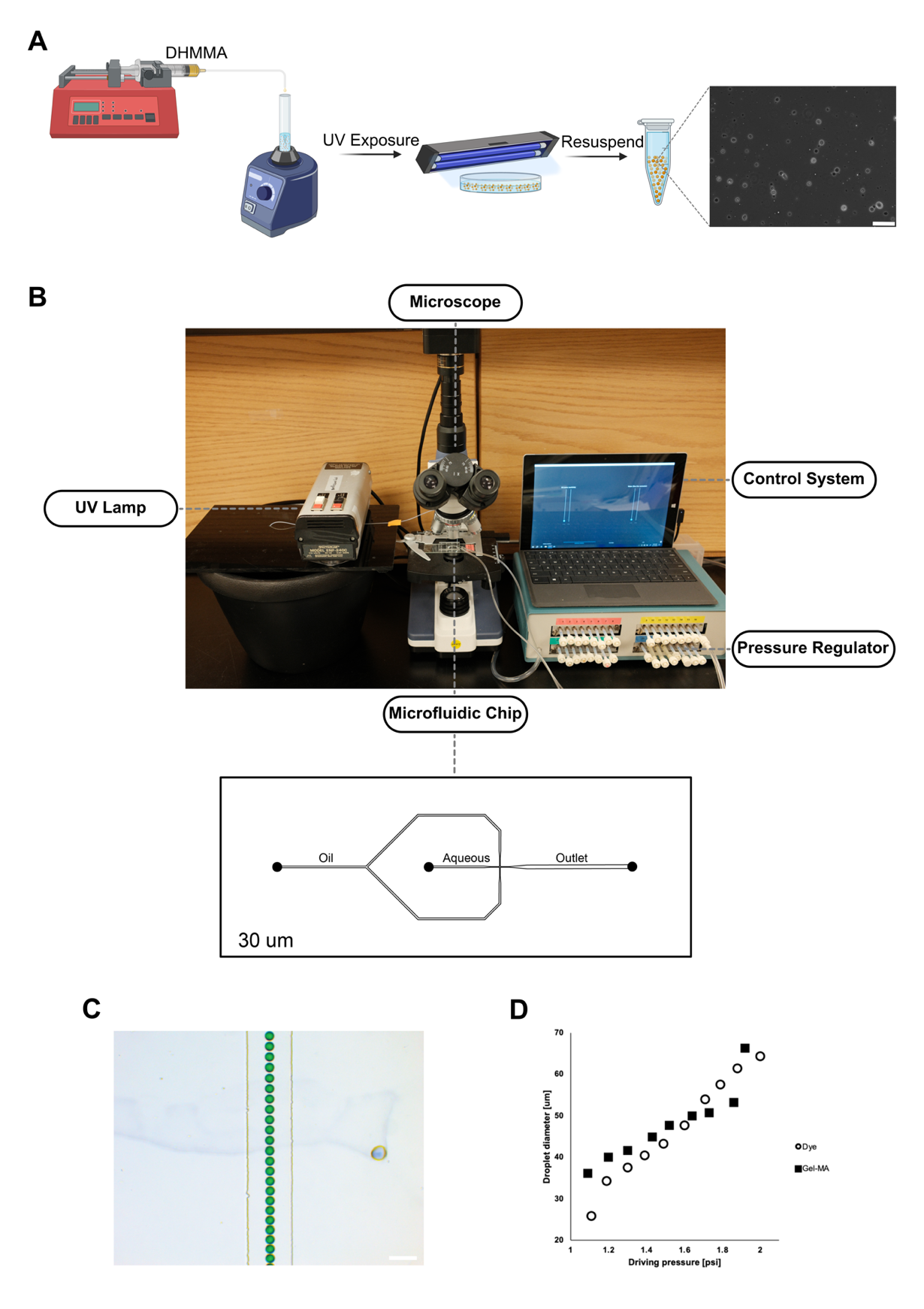


**Figure S2.** Microparticle Generation using Bulk Emulsion and Droplet Microfluidics. **(A)** Generation of crosslinked DHMMA microparticles using bulk emulsion water-in-oil emulsion (Partially created in BioRender <https://BioRender.com/m65b444>). **(B)** Setup for microfluidic droplet generation and schematic of microfluidic design. **(C)** GelMA (with green food dye) droplets in the outlet column. **(D)** Graph illustrating change in GelMA particle size with increasing inlet pressure.


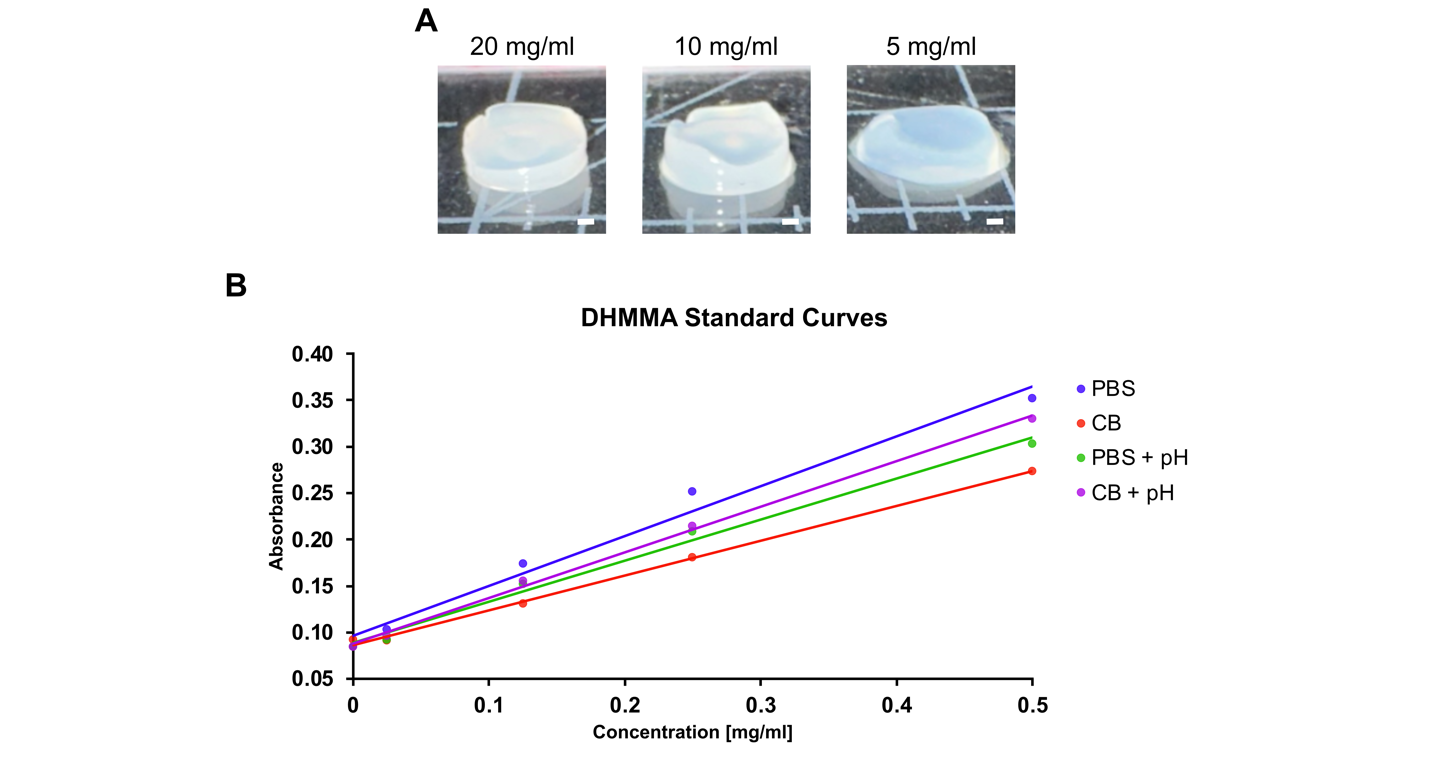


**Figure S3.** **(A)** Crosslinked $\text{DHMM}\text{A}^{\text{PBS}}$ at 20, 10, and 5 mg/ml. **(B)** Standard curve generated for each formulation. Protein concentration determine measured using BCA assay.


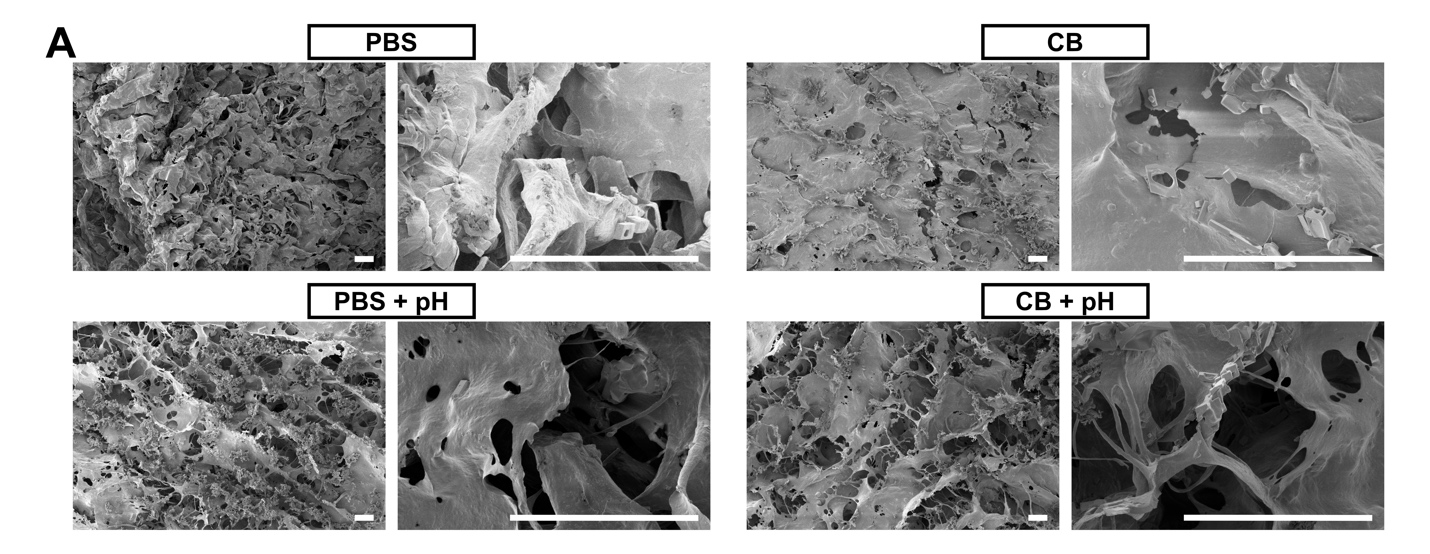


**Figure S4.** **(A)** SEM images of crosslinked DHMMA surface. Scale bar = 50 um.


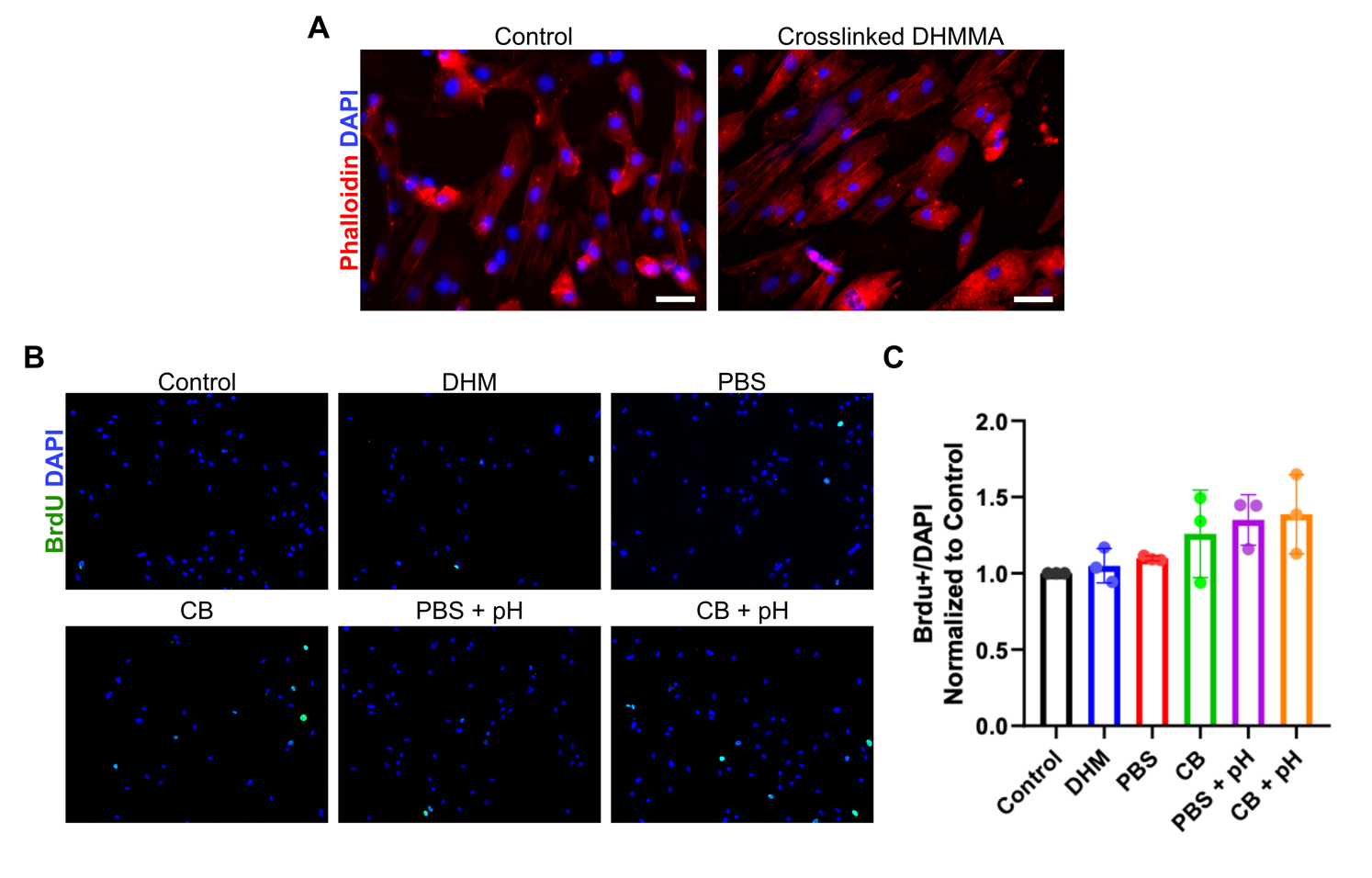


**Figure S5.** DHMMA Bioactivity. **(A)** H9C2 cells cultured on tissue culture plastic (control) or crosslinked $\text{DHMM}\text{A}^{\text{PBS}}$ after 72 h. **(B)** H9C2 cells exposed to soluble DHM/DHMMA for 24 h where cell cycle activity was labeled with BrdU. **(C)** BrdU/DAPI ratio showed the amount of H9C2 cells undergoing cell cycle activity. All data represented as mean ± standard deviation, n = 3. Significance level: ns (p > 0.05) for all conditions.
